# Using Energetic Models to Investigate the Survival and Reproduction of Beaked Whales (family *Ziphiidae*)

**DOI:** 10.1371/journal.pone.0068725

**Published:** 2013-07-17

**Authors:** Leslie F. New, David J. Moretti, Sascha K. Hooker, Daniel P. Costa, Samantha E. Simmons

**Affiliations:** 1 U.S. Marine Mammal Commission, Bethesda, Maryland, United States of America; 2 Naval Undersea Warfare Center, Newport, Rhode Island, United States of America; 3 University of St Andrews, St Andrews, Scotland; 4 University of California, Santa Cruz, California, United States of America; Institut Pluridisciplinaire Hubert Curien, France

## Abstract

Mass stranding of several species of beaked whales (family *Ziphiidae*) associated with exposure to anthropogenic sounds has raised concern for the conservation of these species. However, little is known about the species’ life histories, prey or habitat requirements. Without this knowledge, it becomes difficult to assess the effects of anthropogenic sound, since there is no way to determine whether the disturbance is impacting the species’ physical or environmental requirements. Here we take a bioenergetics approach to address this gap in our knowledge, as the elusive, deep-diving nature of beaked whales has made it hard to study these effects directly. We develop a model for *Ziphiidae* linking feeding energetics to the species’ requirements for survival and reproduction, since these life history traits would be the most likely to be impacted by non-lethal disturbances. Our models suggest that beaked whale reproduction requires energy dense prey, and that poor resource availability would lead to an extension of the inter-calving interval. Further, given current information, it seems that some beaked whale species require relatively high quality habitat in order to meet their requirements for survival and reproduction. As a result, even a small non-lethal disturbance that results in displacement of whales from preferred habitats could potentially impact a population if a significant proportion of that population was affected. We explored the impact of varying ecological parameters and model assumptions on survival and reproduction, and find that calf and fetus survival appear more readily affected than the survival of adult females.

## Introduction

Beaked whales (family *Ziphiidae*) have become a conservation priority following the species’ responses to military sonar and seismic surveys, which range from changes in behavior to stranding and death [1]-[5]. As a result, there is a need to understand how beaked whales respond to disturbance. There have been several studies investigating short-term behavioral responses to sound, e.g., [6]. However, to link such immediate responses to long-term impacts we require improved knowledge of their life history traits, such as survival and reproduction. One approach to better understanding the potential effects of disturbance on marine mammals is to develop a bioenergetics model that integrates short-term behavior, such as foraging, with reproductive output [7], [8]. Development of such a bioenergetics model can improve our understanding of the species’ life history requirements and provide an important framework to understand the response of animal populations to both natural and anthropogenic disturbance.

The six genera and 21 species of beaked whales comprise one of the most diverse and least known families of marine mammals. Some species, such as Hector’s beaked whale (*Mesoplodon peruvianus*) are known from only a few specimens [9], [10]. Necropsies of stranded individuals and historic whaling data, e.g., [11], [12], provide the majority of knowledge on beaked whale biology and physical characteristics (size, weight and sexual dimorphism), and the species’ diets are mostly informed from stomach contents [13]. It is only recently that detailed studies of living individuals and populations have taken place, e.g., [14]-[16]. Due to the development of small data loggers that can acquire information on the whales’ acoustics and their remarkable deep-diving behavior we now have incredibly detailed information on their habitat use, foraging behavior and ecology, e.g., [6], [17], [18]. However, since the geographic distribution of many *Ziphiidae* species are little known and individuals spend only a brief time on the surface, finding and attaching tags to many species of beaked whales remains a challenge [17], [19], [20].

Despite these recent increases in our understanding of *Ziphiidae* movements and foraging behavior, there are still large gaps in our knowledge of their life-history traits. The complications of observing beaked whales *in situ* limit our knowledge of vital rates. It is therefore difficult to estimate population trends, limiting our ability to successfully manage beaked whales or determine the long-term effectiveness of conservation measures [21]. Here, we seek to address many of these issues by synthesizing available data and linking the species’ energetic requirements with knowledge of beaked whale ecology. We develop a general energetics model for *Ziphiidae* to estimate life history traits and threshold requirements for survival and reproduction, as well as their response to any changes in the relevant ecological parameters. By developing a general model for the *Ziphiidae* family, we test known or suggested parameters across beaked whale species, helping to determine the model’s efficacy in identifying gaps and refining the population parameters suggested for the different species; future applications to establish the impacts of disturbance on individual species will then be better informed.

## The Model

Energetics models have previously been used to examine beaked whales’ conservation needs in a spatial context [21]. We use the same class of model to study the species’ life history traits, specifically investigating the percentage of adult females, fetuses, and calves to survive and the relative duration of lactation, given the ecological parameters. In their simplest form energetics models balance energy intake with energy output. Individuals must intake energy to maintain their physical structure, grow and improve condition (i.e., lay down lipid stores). Energy enters the body through feeding, can be mobilized from lipid stores and is output through activity, heat loss and bodily functions [22]. In addition to the daily energetic requirements defined by individual metabolic rate, we are also interested in the resources available for female reproduction (i.e., gestation and lactation), which is another form of energy output.

Accounting for energy intake relies on knowledge of prey species consumed and the energetic content of that prey. Details of prey requirements for parents provisioning offspring are limited [23]. Drent and Daan [24] argue that a parent provisioning its young should not exceed four times their basal metabolic rate. Among marine mammals, differences in the stomach contents of lactating and non-lactating northern fur seals led Perez and Mooney [25] to suggest that provisioning a pup requires a female fur seal to consume 1.6 times as much food as a non-lactating female, and other species are known to increase their prey intake during lactation [26], [27], [28].

### Adult Growth and Maintenance

Given the lack of information for cetaceans we chose to use a conservative estimate of energy intake derived from the allometric relationship between mass (*M,* kg), and biomass ingestion for odontocetes [29]. Therefore, accounting for the percentage of accessible energy (*A*) in the consumed prey, the maximum total adult energy intake is obtained (*E_a_,* kCal day^−1^) using the average energetic content of the whale’s prey (*P,* kCal kg^−1^) (for a list of model parameters see Table 1, while measured parameter values are given in Tables 2–3),
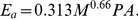
(1)


**Table 1:** The model parameters, their definitions, and the distributions from which parameter values are drawn in the absence of data or biological knowledge.

Parameter	Definition	Distribution
*A*	Assimilation efficiency	*U*(0.60, 0.94)
*P*	Prey energetic content (kJ g^−1^)	*U*(4.5, 9.0)
*L*	Length (cm)	–
*L_b_*	Neonate’s proportion of maternal length at birth (%)	*U*(0.41, 0.54)
*L_w_*	Calf’s proportion of maternal length at weaning (%)	*U*(0.55, 0.86)
*M*	Adult mass (kg)	–
*M_g_*	Mass of the neonate (kg)	–
*M_c_*	Mass of the calf (kg)	–
*A*	Parameter relating length to mass	–
*B*	Parameter relating length to mass	–
*C*	Adult constant for determining active metabolic rate	–
*c_c_*	Calf constant for determining active metabolic rate	–
*Q*	Basel metabolic rate for adults (kCal day^−1^)	–
*Q_c_*	Basel metabolic rate for calves (kCal day^−1^)	–
*p_w_*	Proportion of calf’s daily energy requirements provided by the mother (%)	–
*Q*	Habitat quality	–
*E_a_*	Maximum adult energy intake (kCal)	–
*E_I_*	Actual adult energy intake (kCal)	–
*E_c_*	Daily calf energy requirement (kCal)	–
*E_s_*	Energy stored in lipids in an adult female (kCal)	–
*E_s,l_*	Lower limit of an adult’s energy stores for calf survival (kCal)	*U*(10^4^, 2*cQ*)
*E_s,p_*	Energy in storage at the start of pregnancy (kCal)	*U*(10*E_s,l_,* 20*E_s,l_*)
*E_s,c_*	Energy in storage at the start of lactation (kCal)	*U*(*E_s,p_,* 2*E_s,p_*)
*E_r_*	Energy required by the calf, from the mother (kCal)	–
*E_m_*	Actual maternal contribution to the calf’s daily energy requirements (kCal)	–
*E_j_*	Calf’s contribution to their own energy requirements (kCal)	–
*G*	Total energetic cost of gestation (kCal)	–
*g_t_*	Length of gestation (days)	–
*W*	Time to weaning (days)	–

**Table 2:** The length (*L*), group size (*N*), prey and prey energetic values (*P*) for the 21 beaked whale species, with references.

Species	*L* (m)	*N*	Prey Items	*P* (kJ g^−1^)^a^
*Hyperoodon ampullatus*	6.5[21]	7[16]	*Gonatus* sp.[21]	7.8[21]
*H. planifrons*	6.5[60]	5[61]	*Gonatus* sp.[62]	7.8[21]
*Mesoplodon densirostris*	4.6[63]	9[64]	Hake[13]	5[65]
*M. hectori*	4.4[66]	6	pelagic squid[66]	–
*M. mirus*	4.8[60]	3[67], [68]	*Teuthowenia* sp., fish[13]	–
*M. europaeus*	4.5[60]	4[69]	fish, crustaceans, squid[13]	–
*M. bidens*	5[63]	8[17]	*Diaphus* sp.[70]	9[65]
*M. grayi*	4.6[60]	7[71]	Hake[13]	5[65]
*M. peruvianus*	3.4[72]	5[10]	Nemipteridae[13]	4.5[73]
*M. bowdoini*	4.3[56]	6	mesopelagic squid	–
*M. traversii*	4.6[63]	6	mesopelagic squid	–
*M. carlhubbsi*	4.9[60]	6	*Gonatus, Onychoteuthis* borealijaponica[74]	7[21], [75]
*M. ginkodens*	4.9[63]	6	mesopelagic squid	–
*M. stejnegeri*	4.8[60]	15[76]	*Gonatus* sp., *Gonatopsis-Berrytuethi* [77]	7.2[21], [75]
*M. layardii*	5.8[60]	5[61]	Squid[78]	–
*M. perrini*	4.4[79]	6	pelagic squid	–
*Indopacetus pacificus*	7[80]	7[80]	Onychoteuthis borealijaponica[81]	6.1[75]
*Ziphius cavirostris*	5.5[60]	5[64]	Gonatus sp.[13]	7.8[21]
*Tasmacetus shepherdi*	6.6[51], [82]	6[83]	*Merluccius hubbsi* [51]	5.3[65]
*Berardius bairdii*	11[32]	10[31]	Pollock[52]	5.7[75]
*B. arnuxii*	7.8[33]	15[33], [84]	Squid	–

a The listed prey items are those for which *P* were available, although many of the whale species consume additional prey [13].

**Table 3:** The gestation time (*g_t_*), calf’s percentage of mother’s length at birth (*L_b_*) and weaning (*L_w_*), and time to weaning (*W*), (i.e., duration of lactation) for all 21 beaked whale species, with references.

Species	*g_t_* (days)^a^	*L_b_* (%)	*L_w_* (%)	*W* (days)^a^
*Hyperoodon ampullatus*	365[41]	0.46[14]	0.86[41]	365[41]
*H. planifrons*	365[41]	0.45[85]	0.56[85]	365[85]
*Mesoplodon densirostris*	365	0.42[63]	0.58*	986*
*M. hectori*	365	0.43[9]	0.56[66]	365
*M. mirus*	365	0.49[63]	–	365
*M. europaeus*	365	0.47[63]	–	365
*M. bidens*	365	0.48[63]	0.77[70] (immature)	365
*M. grayi*	365	0.53[63]	–	365
*M. peruvianus*	365	0.45[9]	0.71[9]	365
*M. bowdoini*	365	0.49[56]	–	365[56]
*M. traversii*	365	–	–	365
*M. carlhubbsi*	365	0.51[74]	–	365[74]
*M. ginkodens*	365	–	–	365
*M. stejnegeri*	365	0.5[86]	0.62[87] (2 year old)	365[77]
*M. layardii*	365	0.48[88]	–	365
*M. perrini*	365	0.47[79]	0.56[79]	365
*Indopacetus* *pacificus*	365	0.48[53]	0.61[53]	365
*Ziphius cavirostris*	365	0.4[89]	0.78[90] (subadult)	365
*Tasmacetus shepherdi*	365	0.46[51]	0.57[37]	365
*Berardius* *bairdii*	515[32]	0.41[38]	0.55[91]	183[43]
*B. arnuxii*	515	0.54[92]	–	183

a Where a value is given with no reference, best biological knowledge was used.

* Claridge D pers. comm.

The large size of beaked whales tends to preclude direct measurements of body mass. However, length data are usually available, so information on *M* can be obtained from length-weight relationships,
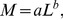
(2)where *L* is length in centimeters (Table 2). Two sets of values for *a* and *b* have been estimated, one for northern bottlenose whales (*Hyperoodon ampullatus*) (*a = *1.3*10^−5^, *b* = 3.07) [30] and one for Baird’s beaked whales (*Berarduis bairdii*) (*a = *6.339*10^−6^, *b = *3.081) [31]. We assign parameter values for species without direct estimates of *a* and *b* based on parsimony with the species’ characteristics. Baird’s beaked whales are the largest species at 10–13 m [32], [33], while the majority of *Ziphiidae* are between 4–7 m in size [34]. Therefore, the parameter values for the northern bottlenose whale, which average 6.5 m in length [21], will generally be more appropriate for these smaller species.

Body mass is also used to estimate base energy expenditure [22],
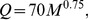
(3)for an adult, where *Q* is the basal metabolic rate (kCal day^−1^). To scale for active metabolism and thus derive the basic energy required for an individual female’s personal growth and maintenance, the value *Q* is multiplied by a constant, *c*, which we set equal to 2.5 [21], [35] (Table 1). Although supported by the literature, the chosen value for *c* is somewhat arbitrary.

### Gestation

In addition to growth and maintenance, energy is required for reproduction. For females, the extra cost is due to gestation (*G,* kCal) and lactation. Gestation costs can be calculated [36] as,
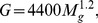
(4)where *M_g_* is the mass of the neonate in kilograms. The length of beaked whale neonates ranges from as little as 1.9 m in Hector’s beaked whale (*Mesoplodon hectori*) [37] to as much as 4.6 m in Baird’s beaked whale [38], so estimates of *M_g_* can be obtained (Eqn. 2).

### Lactation

The cost of lactation depends on the time since parturition and the mother’s provisioning strategy. Since calves are less dependent on their mothers’ milk closer to weaning the simplest assumption is that the proportion of the calf’s daily energy requirements provided by the mother (*p_w_*) declines linearly with time, from 100% at birth to 0% at weaning (*W*),
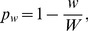
(5)where *w* is the age of a nursing calf in days. We also investigated approximates of asymptotic, step and sigmoidal functions for maternal provisioning of the calf.

As with adult females, the metabolic requirement of calves can be calculated using body mass [39], so the total energy required by the calf from birth to weaning (*E_c_*, kCal) is,
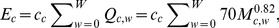
(6)where *M_c,w_* is calf mass at age *w,* based on growth curves, e.g., [32] (Eqn. 2), *Q_c,w_* is the basal metabolic rate for a calf of age *w*, and *c_c_* is the constant used to account for the calf’s active metabolism (Table 1). This value will be higher than the adult metabolic constant (*c*), due to calves' rapid growth from birth to weaning. In addition, since calves have greater metabolic requirements than adults, the slope (0.82) of the allometric relationship is also adjusted accordingly [40]. The mother’s total required contribution (*E_r_*) to *E_c_* is dependent on her provisioning strategy and is calculated as,



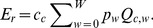
(7)The remainder of the energy required by the calf to reach weaning (*E_j_,* kCal) is assumed to come from the calf foraging on the same prey species as the adults, so that,
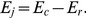
(8)


In the absence of other information, weaning for beaked whales, and other odontocetes, is often assumed to take one year [41], although associations between mothers and calves have been known to last longer [42]. We assume that normal time to weaning takes one year, unless there is evidence to the contrary [32], [43].

### Energy Stores

**Figure 1:**
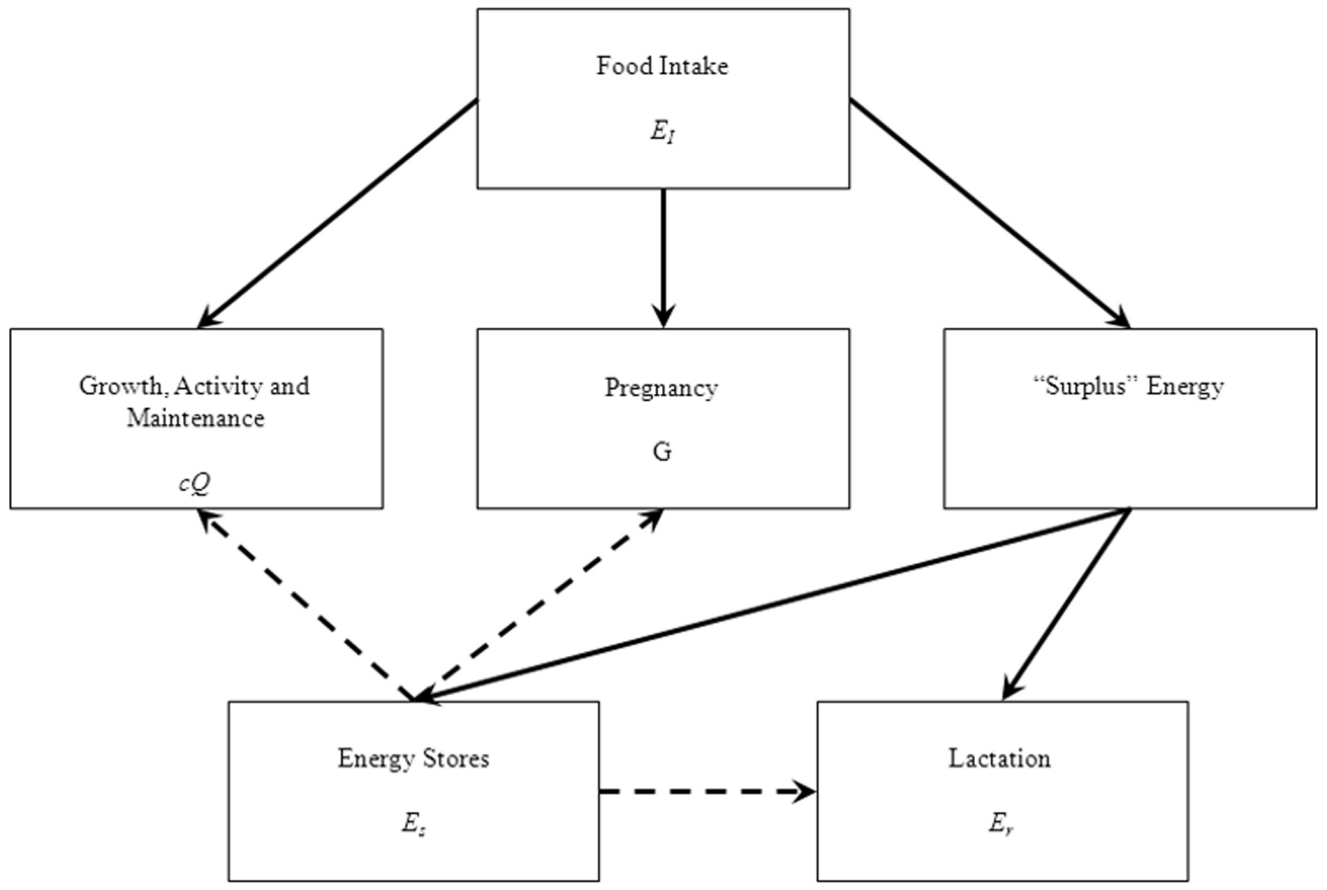
A flow chart representing the movement of energy in adult female beaked whales. Accepting that energy transfer from one state to another is imperfect, the largest proportion of energy will go to growth, activity and maintenance, then pregnancy. Any “surplus” energy (energy not used for *cQ* or *G*) with either go to lactation (*E_r_*) or storage. The relationships between energy stores and lactation, growth and maintenance are dashed lines, since these links will only come into play when food intake can’t supply *E_r_*, *cQ* or *G*.

**Figure 2:**
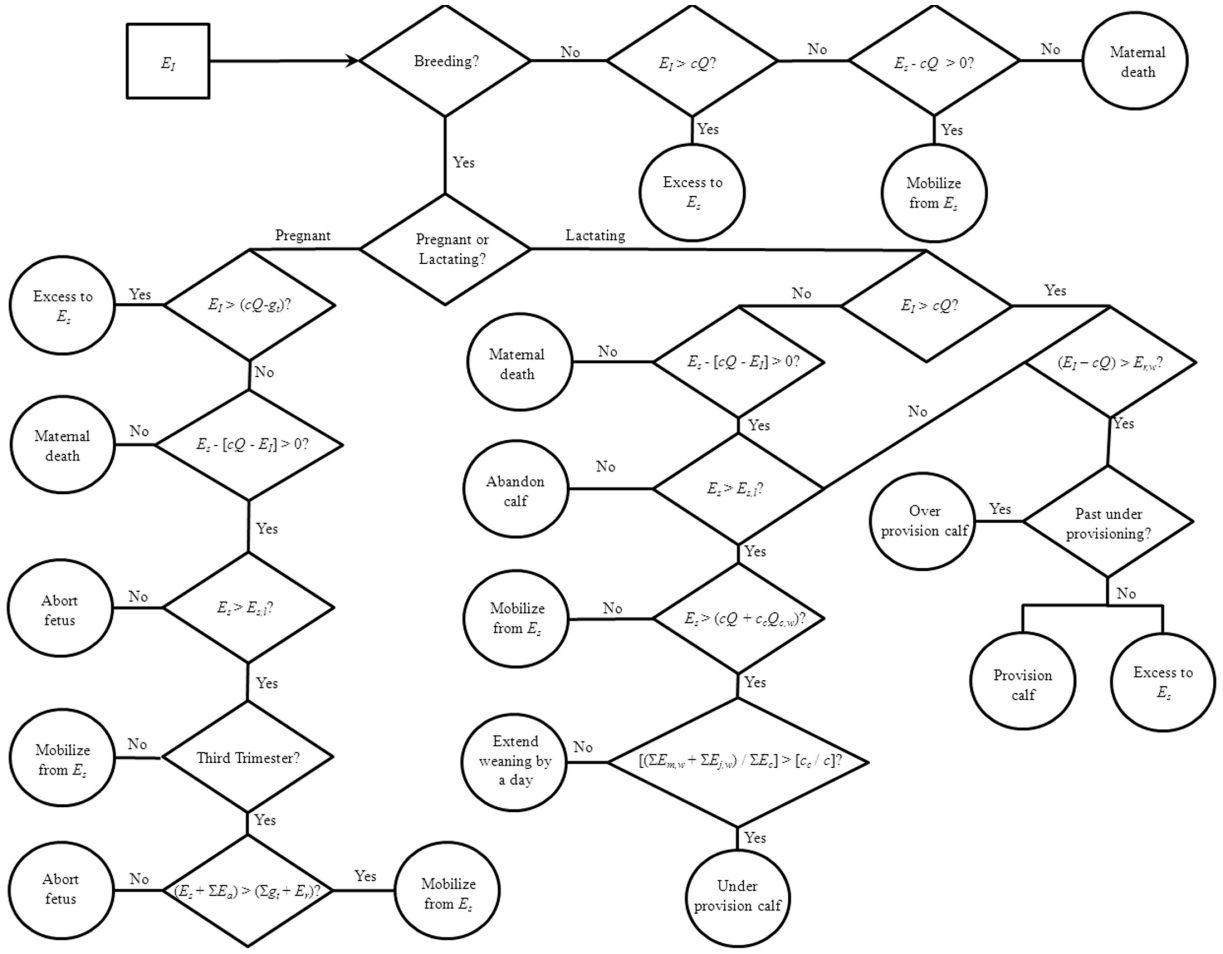
A decision tree representing one time step (a day) in the model simulations for the energy budget of an adult female beaked whale. While all individuals ingest energy (*E_I_*) at the start of a time step, how that energy is used and partitioned depends on the female’s reproductive status and her energy stores (*E_s_*). The square is the starting point of the decision process, diamonds represent decision points and circles are a possible outcome for the time step, given the previous decisions.

Beaked whales accumulate lipid stores prior to and between (recovery phase) reproductive events (capital breeding), as well as taking in energy throughout the breeding cycle (income breeding) [44]. Therefore, a proportion of the energy mobilized for reproduction is derived from lipid stores (*E_s_,* kCal). These stores will be laid down during gestation and anytime the females’ energy intake is greater than that required for reproduction, growth and maintenance (this is inherent in our calculations of *E_a_*). The relationships between energy intake, growth and maintenance, lipid stores and reproduction can be summarized in an energy flow diagram (Fig. 1) (which follows that proposed by Lockyer [45]) and a decision tree (Fig. 2). An important aspect of this relationship is that as prey resources become less available, due to natural variation or to disturbance, the ability of the female to take in excess energy is reduced. This has two outcomes. First, during reproduction, and especially lactation when her energy requirements are greatest, the female will either reduce the amount of energy she transfers to her calf or she will have to utilize her stored energy reserves. This will prolong the duration of lactation or the post-reproduction recovery period, since the female will have to recoup the resources she expended on the calf. Either way this will increase the inter-calf interval and have a direct effect on demography. Second, there will also be a threshold at which there is insufficient energy for the female to do anything other than maintain herself and she will have to terminate lactation or gestation. This energetic threshold is higher (i.e., female requires greater surplus energy) for lactation as it is significantly more costly than gestation.

The energy contributed to *E_s_* will vary with females’ actual energy intake (*E_I_*). If *E_I_*<*cQ,* then females will metabolize energy from their lipid stores. We assume adult females prioritize their own survival and this may result in a reduction in the energy available to a calf, which may lead to an increased inter-calving interval by two possible mechanisms. First, if a female has a calf, low *E_s_* would result in her daily contribution to the calf’s energy needs (*E_m,w_*) being lower than the calf’s daily requirement (*E_r,w_*). Lactation is assumed to be extended by a day if the reduction in *E_m,w_* results in the total energy intake by the calf on day *w* being less than *cQ_c,w_*, since *c_c_*>*c* this would indicate that calf did not receive enough energy for growth. If this disruption to calf growth occurs regularly, then the calf will take longer to wean, extending the inter-calf interval. Furthermore, if beaked whales have a fixed or primary breeding season, then delayed weaning could inhibit the female from becoming pregnant, thus extending the inter-calf interval. Second, if the female is pregnant, we assume that from the third trimester, the fetus will be aborted if the adult’s combined energy reserves and maximum potential energy intake are less than the total amount of energy needed to bring the fetus to term and wean the calf. If the fetus is aborted the adult’s ‘surplus’ energy will be placed in storage and another pregnancy will occur at the next breeding opportunity.

## Simulations

We accounted for heterogeneity by drawing an individual female’s length (*L_i_*) from a normal distribution centered on the average length of a mature female for each species (Table 2),
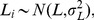
(9)where the variance (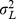) was tuned to ensure that *L_i_* did not take biologically unrealistic values. Since we were interested in investigating beaked whale reproduction, the simulation limited female reproductive status to pregnant or lactating (i.e., breeding), which was determined through a Bernoulli distribution where *p* = 0.5. Variability in energy intake was included by drawing the value for actual daily adult female energy intake, *E_I_,* from a normal distribution truncated between zero and *E_a_,*

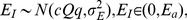
(10)where 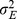 is equal to the variance of the Ea values within a species’ group and q represents habitat quality. Since knowledge of beaked whale habitat is limited, q can be representative of either greater prey density, or the availability of prey of greater energetic value. Values of q greater than one indicate that a female’s average energy intake will be greater than required for personal growth and maintenance (cQ). In contrast, values of q less than one indicate that, on average, females’ daily energy intake will be less than daily energy requirements for growth and maintenance, which would require them to metabolize energy from their lipid stores.

Each female has energy reserves (i.e., lipid stores) at the start of the simulation (for a list of the parameters and their distributions see Table 1), the value of which is dependent on her reproductive status. Females are assumed to have a minimal threshold of energy storage (*E_s,l_*) drawn from *U*(10^4^, 2*cQ*). If this value is reached lactating females will prioritize their own survival by abandoning their calves, which results in calf mortality. The lower limit minimizes the probability of calf abandonment, since it is less than *cQ* for all species, while the upper limit requires females to maintain at least two days’ worth of energy in storage. Energy reserves at the start of gestation (*E_s,p_*) should always be higher than *E_s,l_* if a fetus is to be brought to term. Therefore, *E_s,p_* is drawn from *U*(10*E_s,l_*, 20*E_s,l_*), giving the females a week and a half to three weeks of reserves at the start of gestation. Finally, lactation requires greater energy inputs than gestation, so the initial energy reserves for a female with a calf (*E_s,c_*) are drawn from *U*(*E_s,p_*, 2*E_s,p_*). What we refer to as lipid stores or energy reserves includes only those tissues that can be metabolized by the female and therefore excludes structural tissue. As a result, *E_s_* can be equal to zero, although this is assumed to cause the death of the female and her calf or fetus.

To incorporate variation in calf size we calculated calf length at birth and weaning as a proportion of mother’s length (*L_b_* and *L_w_*, respectively, Table 3). Calves will therefore vary in size with the females, although the proportion of the length of the mother is constant within each species. For each species, the number of individuals in the simulation was equal to an observed group size for each species (Table 2), so that we can assume individuals are affected equally by habitat quality (*q*). This assumption does not affect the current model outputs, but can be relaxed to allow for spatial or temporal variation in *q*, when dealing with larger populations and individual species in more detail.

The simulation starts at either breeding or birth, assuming the two events occur in relative synchrony. For pregnant females, the cost of gestation is assumed to be unavoidable and equally spread over the gestation period, so the daily energy requirements are equal to,
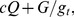
(11)where *g_t_* is the gestation time. *‘*Surplus’ energy can be placed in storage, or storage can be metabolized to meet a female’s energy needs. For females with calves, any ‘surplus’ energy ingested is placed into milk production; any additional energy required by the calf is metabolized from *E_s_.* If there is energy remaining after providing *E_r,w_*, it will be placed in *E_s_*. Since adult females are assumed to prioritize their own survival, a female forced to metabolize her energy stores to meet her own energy demands will only supply energy to the calf if her reserves are greater than,
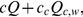
(12)and will otherwise under-provision her calf. Should a female’s energy reserves fall below Es,l, she will abandon her calf. After the abandonment, all ‘surplus’ energy will go to the female’s stores and she will become pregnant again at the next breeding opportunity. This assumes that the cost of gestation is low in comparison to the fitness cost of missing a breeding opportunity. However, if the gap between calf abandonment and breeding opportunity is brief, it is possible that a female will choose to delay pregnancy in order to increase her condition and the chance of successful future reproduction.

We used the model structure described above (Fig. 2) to run simulations of beaked whale energy budgets in the statistical programming language R [46]. The focus of the simulations was to investigate the different possible functional forms for maternal calf provisioning and determine the general energetic requirements of beaked whales for female, calf and fetus survival and hence reproduction.

### Functional Forms for Maternal Provisioning

**Figure 3:**
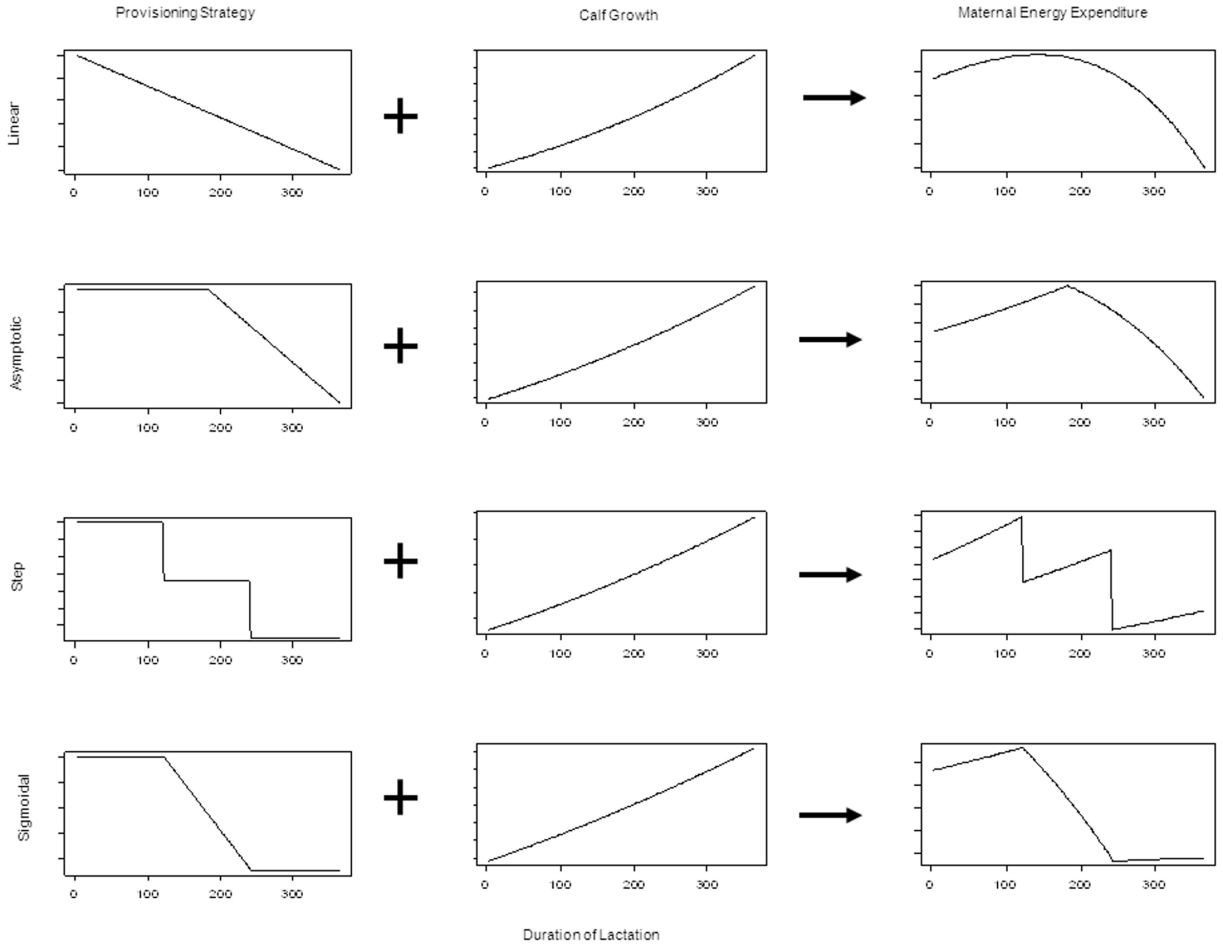
Four maternal provisioning strategies (left column), when combined with calf growth (middle column) give the actual energy expenditure required by the mother each day (right column) over the duration of lactation. The four provisioning strategies are linear (top row), asymptotic (second row), step function (third row) and sigmoidal (bottom row).

Four different functional forms of declining maternal investment strategies were considered; a) the linear relationship in Eqn. 5, b) an approximated asymptote, c) a step function and d) an approximated sigmoidal (Fig. 3). The value of *E_r,w_* will depend on the stage of lactation and calf size. Since beaked whales grow most rapidly from birth to weaning, we treat this relationship as linear, acknowledging that the rate of growth would decrease after separation from the mother. Therefore, we have almost linear growth in calf mass over the lactation period (Eqn. 2). The growth curve and provisioning curve, when combined, describe the mother’s expected required energy contribution to calf growth (Fig. 3). Of the four possible provisioning strategies, only the linear function results in biologically reasonable estimates of the provisioning curve, based on the fat content of milk [47]. We do not suggest that the linear assumption is the actual maternal provisioning strategy for beaked whales, especially given the lack of evidence for this functional form in other cetaceans. However, since the output of the linear assumption is the most biologically realistic, we use this provisioning strategy when investigating survival and reproduction.

### Beaked Whale Energetics

We applied our model to all 21 species of beaked whales. Parameter values for each species were taken from the literature where possible (Tables 2 and 3), but there were some limitations. In many cases, the average energetic value of prey (*P*) was only known for one or two of the species consumed. Furthermore, not all parameter values were available for each species, with *P* and the calf’s proportion of the mother’s length at birth (*L_b_*) and weaning (*L_w_*) being the three values most commonly missing. When unknown, these parameters were drawn from a uniform distribution whose range was equal to the lowest and highest known values for these parameters, as identified for other beaked whale species (Table 1). In addition, habitat quality is an unknown quantity, and was therefore chosen from a *U*(0.5,2) distribution (Table 1). This range allows habit quality to vary from extremely poor to superlative. Lastly, the percentage of accessible energy (*A*) available to beaked whales after the consumption of prey is also unknown. This parameter value was drawn from a *U*(0.60,0.94) distribution (Table 1), since these are upper and lower estimates available for assimilation efficiency of squid for other species, e.g., [48], [49]. The lower limit of 0.60 is conservative in this case, since the actual assimilation efficiency for squid is likely to be 70–80% [50]. However, although assimilation efficiencies are used to inform the limits of the distribution for *A*, accessible energy also encompasses metabolic efficiency (energy available for metabolism after fecal and urinary energy loss is removed), so the proportion of energy that can be utilized by the beaked whales will be lower than implied by assimilation efficiency alone. We ran 1000 simulations for each species, recording the simulated values for the parameters drawn from distributions (Table 1), time to weaning and the percentage of adults, calves and fetuses in each group to survive.

Some parameter values will never allow for adult survival. Therefore, starting with *E_s,c_* we identified the lowest value for which adult survival is greater than zero and discarded the results from any simulation whose *E_s,c_* value was below this threshold. We then did the same for *E_s,p_*, *E_s,l_*, *A* and *q,* in that order. However, for *E_s,l_* we identified the maximum, rather than the minimum, value at which survival was greater than zero, since higher values indicate that females are more sensitive to their own need for survival. This left us with the outputs from 7–76% of the simulations and provided us with lower limits on these parameter values for which survival is never possible for the adult females (Table 4). The species’ response to these parameters can be ascertained from the number of simulation results remaining after the discard process had taken place: the fewer the remaining simulation results, the more affected the species is by parameter variability.

**Table 4:** The lower bound for parameters *E_s,p_, E_s,c_, A* and *q*, below which adult survival does not occur, the upper bound for *E_s,l_,* above which survival does not occur, and the percentage of the simulations retained (%).

Whale	*E_s,p_* (*10^5^)	*E_s,c_* (*10^5^)	*E_s,l_* (*10^5^)	*A*	*q*	%
*Hyperoodon ampullatus*	1.89	1.40	3.73	0.60	0.93	72
*H. planifrons*	1.83	1.24	3.74	0.60	0.94	70
*Mesoplodon densirostris*	1.18	1.13	1.17	0.88	1.12	11
*M. hectori*	1.27	1.09	1.79	0.60	0.86	74
*M. mirus*	1.16	1.15	1.89	0.60	0.94	70
*M. europaeus*	1.67	1.21	2.22	0.60	0.89	76
*M. bidens*	1.83	1.36	2.59	0.60	0.88	76
*M. grayi*	2.17	1.46	1.29	0.85	1.05	18
*M. peruvianus*	4.63	3.35	6.55	0.86	1.28	7
*M. bowdoini*	1.86	1.39	1.77	0.60	0.95	68
*M. traversii*	1.49	1.19	2.09	0.60	0.91	73
*M. carlhubbsi*	1.47	1.08	1.80	0.60	0.97	67
*M. ginkodens*	1.42	1.16	2.13	0.60	0.89	72
*M. stejnegeri*	1.40	1.08	1.89	0.60	0.87	75
*M. layardii*	1.86	1.46	3.17	0.60	0.97	70
*M. perrini*	1.79	1.28	1.75	0.60	0.94	74
*Indopacetus* *pacificus*	2.02	1.47	3.47	0.75	1.01	38
*Ziphius cavirostris*	1.68	1.32	2.65	0.60	0.89	75
*Tasmacetus* *shepherdi*	2.97	1.70	2.54	0.87	1.13	12
*Berardius* *bairdii*	2.28	2.02	5.18	0.79	0.90	34
*B. arnuxii*	1.08	1.08	3.68	0.60	0.86	77

**Figure 4:**
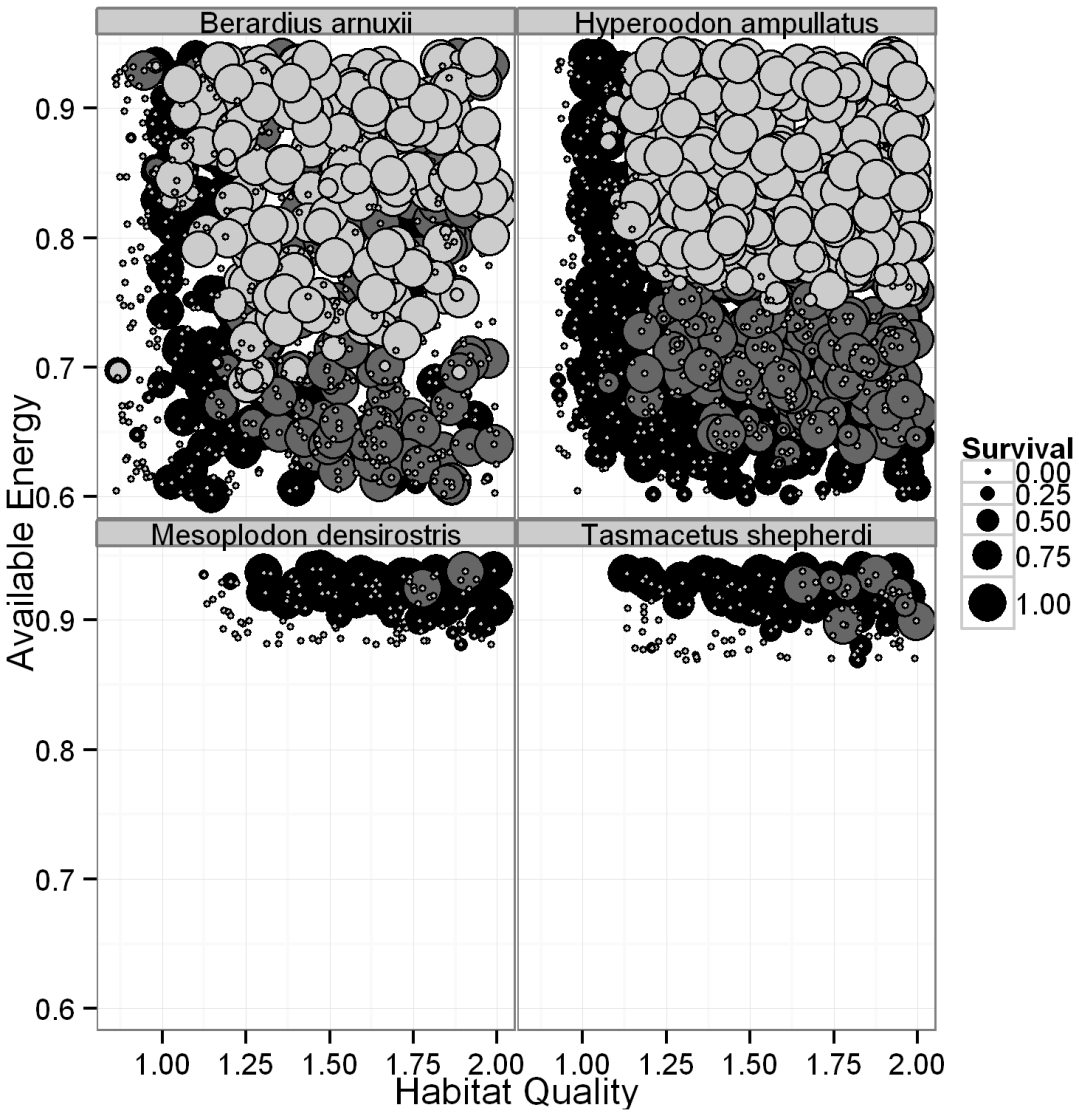
The relationship between habitat quality, available energy and the percentage of adult females (black), calves (dark grey) and fetuses (light grey) in a group to survive, as indicated by the size of the circle. Calves and fetuses can’t survive without their mothers, so adult female survival is not shown when it is equal to that of their offspring. Similarly, if only fetus survival is visible then calf and maternal survival has occurred at the same intensity. Only four species are shown to provide detailed examples of species with high (*B. arnuxii, H. ampullatus*) and low (*M. densirostris, T. shepherdi*) survival and reproduction. Each point is the result from a single simulation.

**Figure 5:**
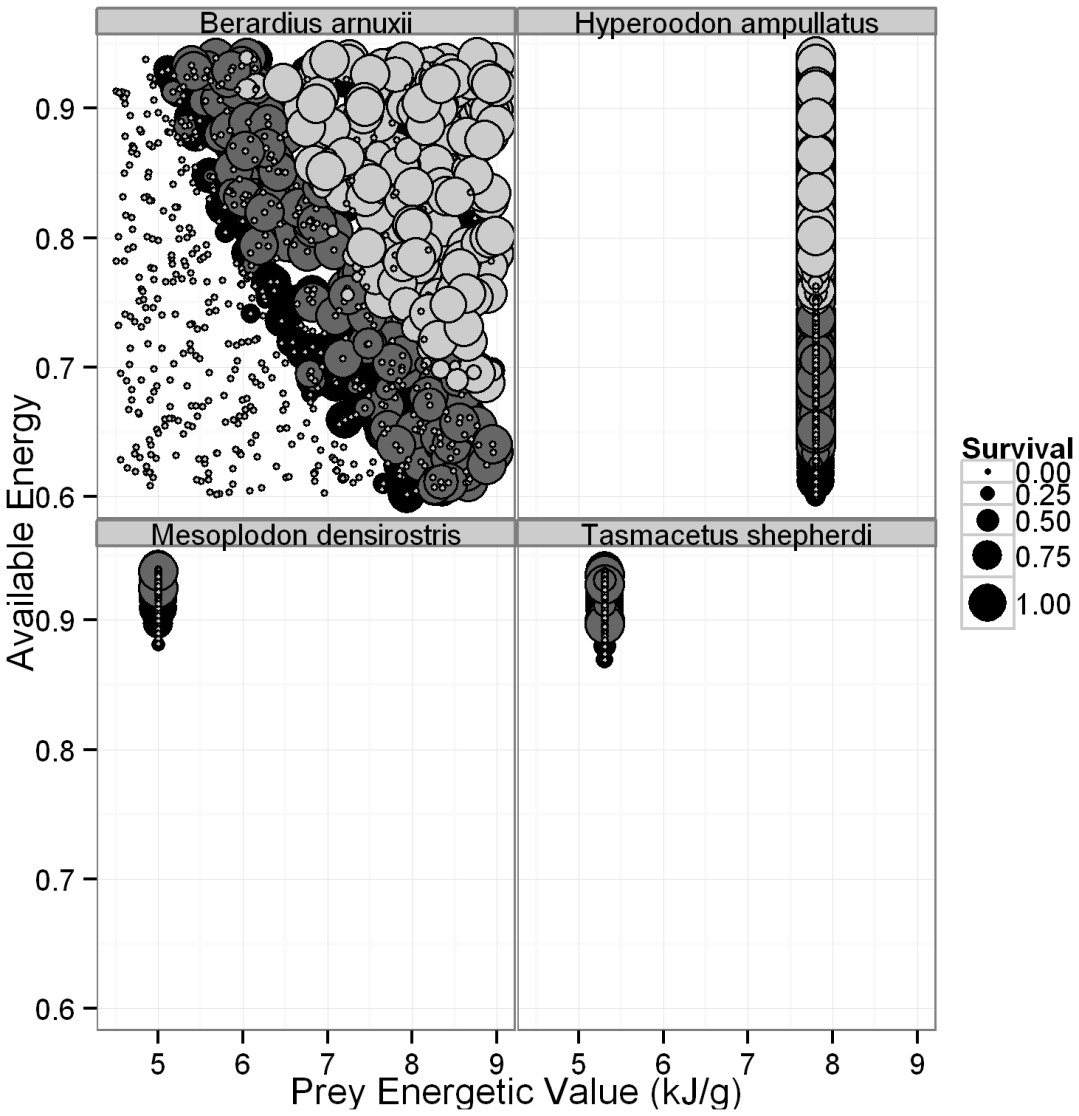
The relationship between the energetic content of prey, available energy and the percentage of adult females (black), calves (dark grey) and fetuses (light grey) in a group to survive, as indicated by the size of the circle. Calves and fetuses can’t survive without their mothers, so adult female survival is not shown when it is equal to that of their offspring. Similarly, if only fetus survival is visible then calf and maternal survival has occurred at the same intensity. Only four species are shown to provide detailed examples of species with high (*B. arnuxii, H. ampullatus*) and low (*M. densirostris, T. shepherdi*) survival and reproduction. Each point is the result from a single simulation.

The simulations resulted in certain patterns across multiple species of beaked whale. For many species (Figs. 4, S1), there is a distinct parameter space in which adults, calves and fetuses will all survive, usually consisting of good habitat quality (*q*) and mid- to high-accessible energy. Unsurprisingly, there is an inverse relationship between the energetic content of the whales’ prey (*P*) and their accessible energy (*A*), with survival at low *P* values requiring high *A* (Figs. 5, S2). For all species, adults survive, but do not produce offspring when *A, q* and *P* are low. Calves start to survive at slightly higher values of these parameters, and fetuses are the most sensitive, requiring good quality habitat and mid- to high values of *A* in order to reach parturition (Figs. 4–5, S1–S2).

**Figure 6:**
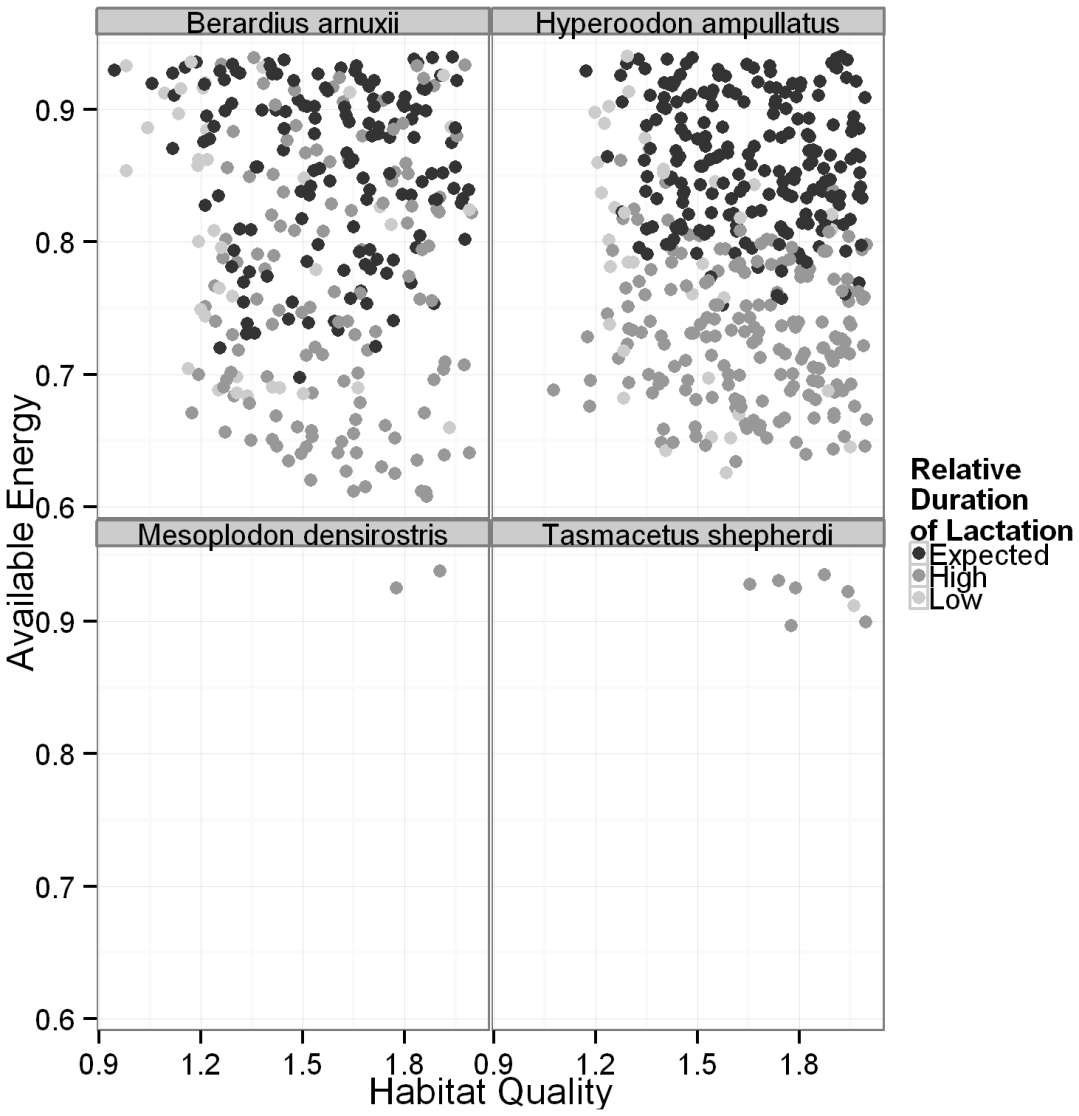
The relationship between available energy, habitat quality and the relative duration of lactation. Light grey points indicate that the relative duration of lactation was less than expected, which means that not all calves survived to weaning. Black points indicate that the duration of lactation was equal to the assumed value and dark grey points indicate that the duration of lactation was longer than expected. Only four species are shown to provide detailed examples of species with high (*B. arnuxii, H. ampullatus*) and low (*M. densirostris, T. shepherdi*) survival and reproduction. Each point is the result from a single simulation.

**Figure 7:**
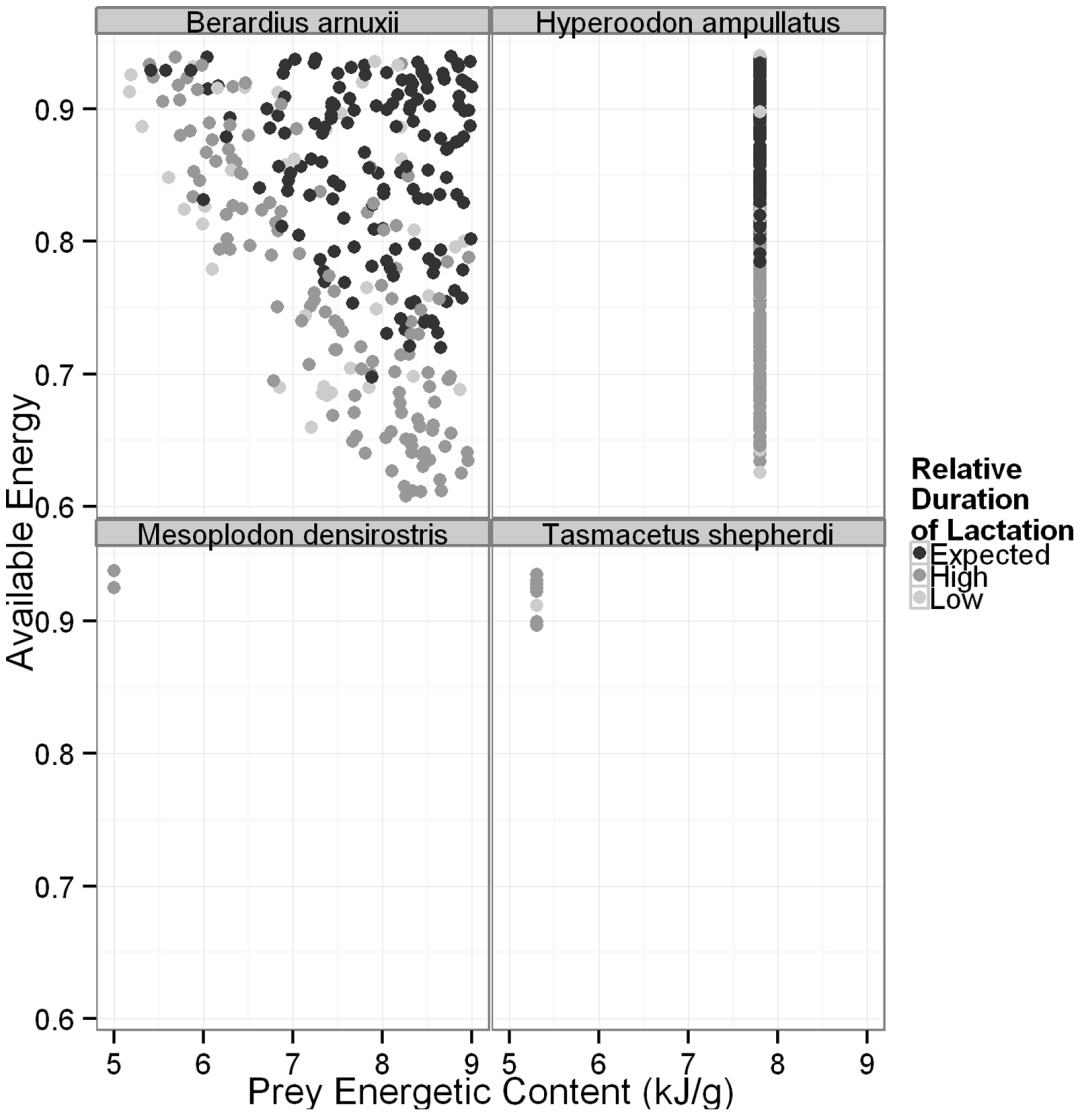
The relationship between available energy, prey energetic content and the relative duration of lactation. Light grey points indicate that the relative duration of lactation was less than expected, which means that not all calves survived to weaning. Black points indicate that the duration of lactation was equal to the assumed value and dark grey points indicate that the duration of lactation was longer than expected. Only four species are shown to provide detailed examples of species with high (*B. arnuxii, H. ampullatus*) and low (*M. densirostris, T. shepherdi*) survival and reproduction. Each point is the result from a single simulation.

High offspring mortality, fetal and calf, will increase the interval between successful calving events. The inter-calving interval can also be extended if a female is required to suckle her calf past the expected weaning time (*W*). This is assumed to delay implantation of another fetus, since pregnancy and lactation are treated as mutually exclusive in order to minimize the energetic burden on the mother. Although the two mechanisms for increasing inter-calving interval are very different, both will result in a decrease in a female’s lifetime reproductive output and are therefore of interest when examining the life history traits of *Ziphiidae*. When the simulated average duration of lactation for the population is less than *W* (Table 3, Figs. 6–7, S3–S4), some proportion of the females abandoned their calves prior to weaning. A simulated value of the average duration of lactation greater than *W* indicates that females struggled to provide sufficient energy to their calves to allow for growth, but were not energetically stressed to the point of calf abandonment. The average duration of lactation for the population was most strongly affected by our assumptions regarding accessible energy, requiring higher values of *A* in order for the average duration of lactation derived from the simulations to be equal to the expected time of weaning (*W*). Habitat quality had less of an impact, although survival of calves was generally rare when *q*<1.2 (Figs. 6, S3). A combination of high *A* and *P* also resulted in the average duration of lactation from these simulations being equal to *W* (Fig. 7, S4).

In our model for six species, Blainvilles, Grey’s (*M. greyi*), pygmy (*M. peruvianus*), Longman’s (*Indopacetus pacificus*), Shepherd’s (*Tasmacetus shepherdi*) and Baird’s beaked whales, adult survival was rare; calf and fetus survival even more so (Figs. 4–5, S1–S2). The duration of lactation was also extended in each of these species, and for pygmy beaked whales, no calves ever survived (Figs. 6–7, S3–S4). These six species all had the greatest number of discarded simulations and were the only ones where the lower limit on *A* is not only greater than 0.60, but in all but two cases was also greater than a less conservative value of 0.80, and all were greater than 0.70 [50] (Table 4). They were therefore more affected by the background parameter variability, since their ‘known’ parameters place them on the knife edge of survival. Of these species Blainville’s, Grey’s, Shepherd’s and pygmy beaked whales had the highest limits on *q* and the lowest estimated energetic content of prey, all with values ≤5.3 kJ g^−1^ (Tables 2 and 4). For species where *P* was unknown, adults rarely survived when energetic content was <5 kJ g^−1^, even at high values of *A* (Figs. 5, S2). The sensitivity of Longman’s and Baird’s beaked whales likely resulted from their large size, making it difficult for the females to build and maintain the required energy reserves for both gestation and lactation. This was especially pronounced in Baird’s beaked whale, which has an estimated six months from birth to weaning [43], requiring high immediate energy input from the mothers.

## Discussion

By combining our knowledge of beaked whales across different species in the family *Ziphiidae* we were able to construct a detailed mathematical model exploring the species’ energetics, survival and reproduction. Modeling all 21 species enabled us to assess our model more fully, helping us to understand the relationship between our assumptions and parameter values over a range of biological inputs that would not be apparent if each species was studied in isolation. Our model takes advantage of current biological knowledge and is flexible enough to incorporate new information as it becomes available. Larger population sizes and variability in habitat over space and time also could readily be incorporated into the model framework if investigating individual species in greater detail.

Despite the uncertainty around some parameter values, by testing a variety of different assumptions we obtained a measure of biological sensitivity to these parameters and were able to determine a range of conditions over which beaked whale survival and reproduction could occur. Combined, this information will help guide future research. Of particular interest are the population consequences of impaired foraging, whether caused by behavioral change or habitat displacement in response to environmental or anthropogenic disturbance. Key data needs include more comprehensive determination of prey species and more accurate determination of reproductive parameters, such as the duration of gestation and lactation, as the model results are particularly affected by these assumptions. While different assumptions for the duration of gestation and lactation can affect the percentage of females, calves and fetus to survive, as well as relative duration of lactation, the relative relationship between the species, their response to parameter variability and the observed patterns in survival and reproduction remained unchanged.

Our simulations accounted for the survival and reproduction of more than two-thirds of the beaked whale species, giving us reasonable confidence in our underlying model. For the six species modeled that regularly failed to survive or reproduce, there may be inaccuracies in the underlying parameters. The main driver of depressed survival and reproduction for these species is the apparently low average energetic content of the beaked whales’ prey. The values for *P* were based on stomach content data, which is often biased or unrepresentative of the whales’ actual diets [13]. For all six of these species, fish formed the majority of the prey items [13], [51], [52], so our results imply that the whales are consuming more energy dense prey than is recorded in the stomach contents in order to persist.

The other driver of low survival and reproduction appeared to be tied to the duration of lactation. Baird’s beaked whale is estimated to wean its young in six months [43], which results in a high daily cost of lactation because of the compressed time to calf independence. Although the gestation period is longer than that of other beaked whales [32], the low *W* meant that female Baird’s beaked whales in our simulations could not ingest enough energy to maintain themselves, grow the fetus and build lipid stores for lactation prior to parturition, and therefore aborted their fetuses. There is some evidence for alloparental care in this species [31], which is not accounted for in our models and may help increase a calf’s independent foraging faster than currently assumed. Longman’s beaked whales face a similar energetic challenge, due to birthing large calves that are 61% of maternal body size at weaning [53]. Therefore, even though they have a longer duration of lactation than Baird’s beaked whales, they apparently suffer similar difficulties in accumulating the energy required for themselves, the fetus and the calf during gestation. Interestingly, in our simulations Longman’s beaked whales did not survive when habitat quality, *q*<1.01, implying that despite a relatively high energetic prey content (*P*>5.3 kJ g^−1^), they had difficulty meeting their metabolic requirements.

The lack of successful calf and fetus production, especially in the larger members of the beaked whale family, may also be a result of our assumption of a two year inter-calving interval for most species (the combined duration of gestation and lactation). Although not explicitly modeled, the assumption is inherent in our choices for the distributions used to define maternal energy stores at the start of gestation and lactation (Table 1). Assuming a two year inter-calving interval does not allow for a recovery period in which females could build energy stores in the absence of gestation or lactation, which would increase a female’s probability of successfully birthing and rearing her next calf. To have a recovery period, a female would need to skip mating opportunities, which would have functionally the same effect as offspring mortality or increased lactation duration, in terms of increasing the inter-calf interval. Given the possibility of a recovery period and the apparently limited successful reproduction in the larger members of *Ziphiidae,* it is possible that these species may have more of a capital breeding strategy, similar to elephant seals [54] or mysticetes [55]. In general, our results raise questions about the appropriateness of assuming that beaked whale reproduction is similar to that of other odontocetes, as has been done in the past, e.g., [56], and highlight the need for further investigation.

The remaining 15 species of beaked whales survived and reproduced over a wide range of parameter values. Some results, such as the requirement for *q*>1 for successful reproduction are unsurprising, since females who are unable to meet their own energy needs cannot provision a calf or meet the cost of gestation, no matter their breeding strategy. There is an apparent flexibility in adult survival across a wide range of habitat quality, accessible energy and prey energetic contents, but more narrow requirements for successful reproduction. Maternal ability to consume and process energy therefore appears to be the primary driver of these vital rates.

Our results suggest that adult female beaked whales are able to survive, but not reproduce, in times or areas of lower habitat quality, and will extend the duration of lactation in mediocre conditions in order to increase the probability of their own survival, but still reproduce. This is biologically reasonable, since resource availability will not be constant through space and time, females are unlikely to successfully reproduce every breeding cycle, and extended lactation periods have been observed in other species, e.g., [57]. This may have demographic consequences, since the lifetime reproductive output of females will decrease with an increasing inter-calf interval. The apparent flexibility of adult survival, but not reproduction implies that anthropogenic disturbances that cause a consistent, minor reduction in energy intake over an extended period of time could potentially impact reproduction just as strongly as disturbances that completely halt energy acquisition over a shorter period. This has implications for conservation and management, due to industry and military activity in beaked whale habitat, which can have seemingly minor impacts on beaked whale behavior or habitat in the short-term, [4], [6], [58], [59]. The use of the energetics model developed here in combination with more detailed research into individual species could elucidate their specific sensitivity to disturbance and the effects of management and conservation action on that species’ persistence within an area.

## Supporting Information

Figure S1 **The relationship between habitat quality, available energy and the percentage of adult females (black), calves (dark grey) and fetuses (light grey) in the population to survive, as indicated by the size of the circle.** Calves and fetuses can’t survive without their mothers, so adult female survival is not shown when it is equal to that of their offspring. Similarly, if only fetus survival is visible then calf and maternal survival has occurred at the same intensity. Each point is the result from a single simulation.
(TIF)

Figure S2 **The relationship between the energetic content of prey, available energy and the percentage of adult females (black), calves (dark grey) and fetuses (light grey) in the population to survive, as indicated by the size of the circle.** Calves and fetuses can’t survive without their mothers, so adult female survival is not shown when it is equal to that of their offspring. Similarly, if only fetus survival is visible then calf and maternal survival has occurred at the same intensity. Each point is the result from a single simulation.
(TIF)

Figure S3 **The relationship between available energy, habitat quality and the relative duration of lactation.** Light grey dots indicate that the relative duration of lactation was less than expected, which means that not all calves survived to weaning. Black dots indicate that the duration of lactation was equal to the assumed value and dark grey dots indicate that the duration of lactation was longer than expected. *M. peruvianus* is not shown, since no simulation estimated successfully weaned calves. Each point is the result from a single simulation.
(TIF)

Figure S4 **The relationship between available energy, prey energetic content and the relative duration of lactation.** Light grey points indicate that the relative duration of lactation was less than expected, which means that not all calves survived to weaning. Black points indicate that the duration of lactation was equal to the assumed value and dark grey points indicate that the duration of lactation was longer than expected. *M. peruvianus* is not shown, since no simulation estimated successfully weaned calves. Each point is the result from a single simulation.
(TIF)
