# Supplementary figures and images for: Using Energetic Models to Investigate the Survival and Reproduction of Beaked Whales (family Ziphiidae)
Source: PLoS One. 2013 Jul 17;8(7):e68725. doi: 10.1371/journal.pone.0068725 (PMC3714291; doi:10.1371/journal.pone.0068725)

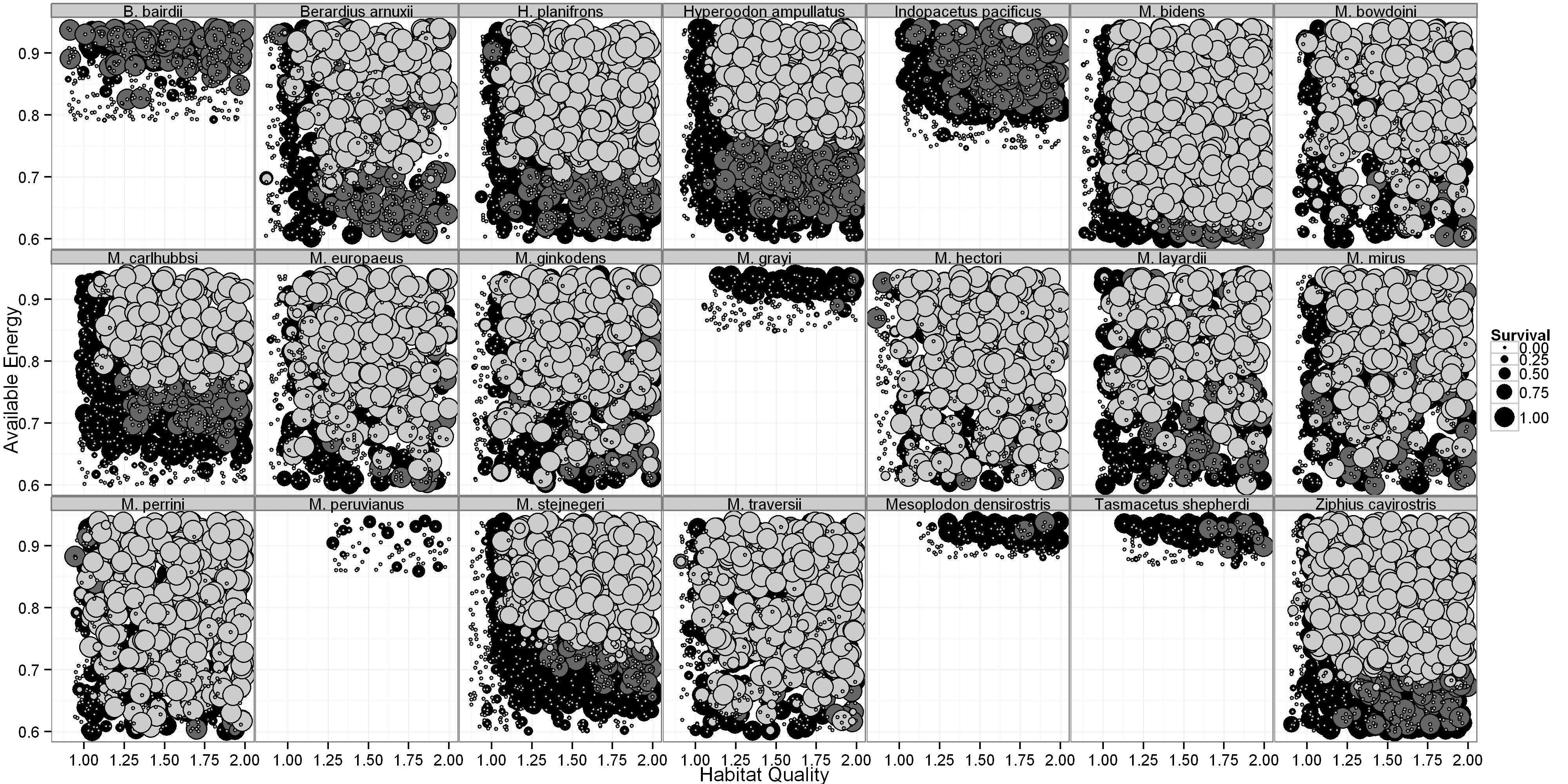

Supplement: Figure S1 — The relationship between habitat quality, available energy and the percentage of adult females (black), calves (dark grey) and fetuses (light grey) in the population to survive, as indicated by the size of the circle. Calves and fetuses can’t survive without their mothers, so adult female survival is not shown when it is equal to that of their offspring. Similarly, if only fetus survival is visible then calf and maternal survival has occurred at the same intensity. Each point is the result from a single simulation. (TIF) [file pone.0068725.s001.tif]

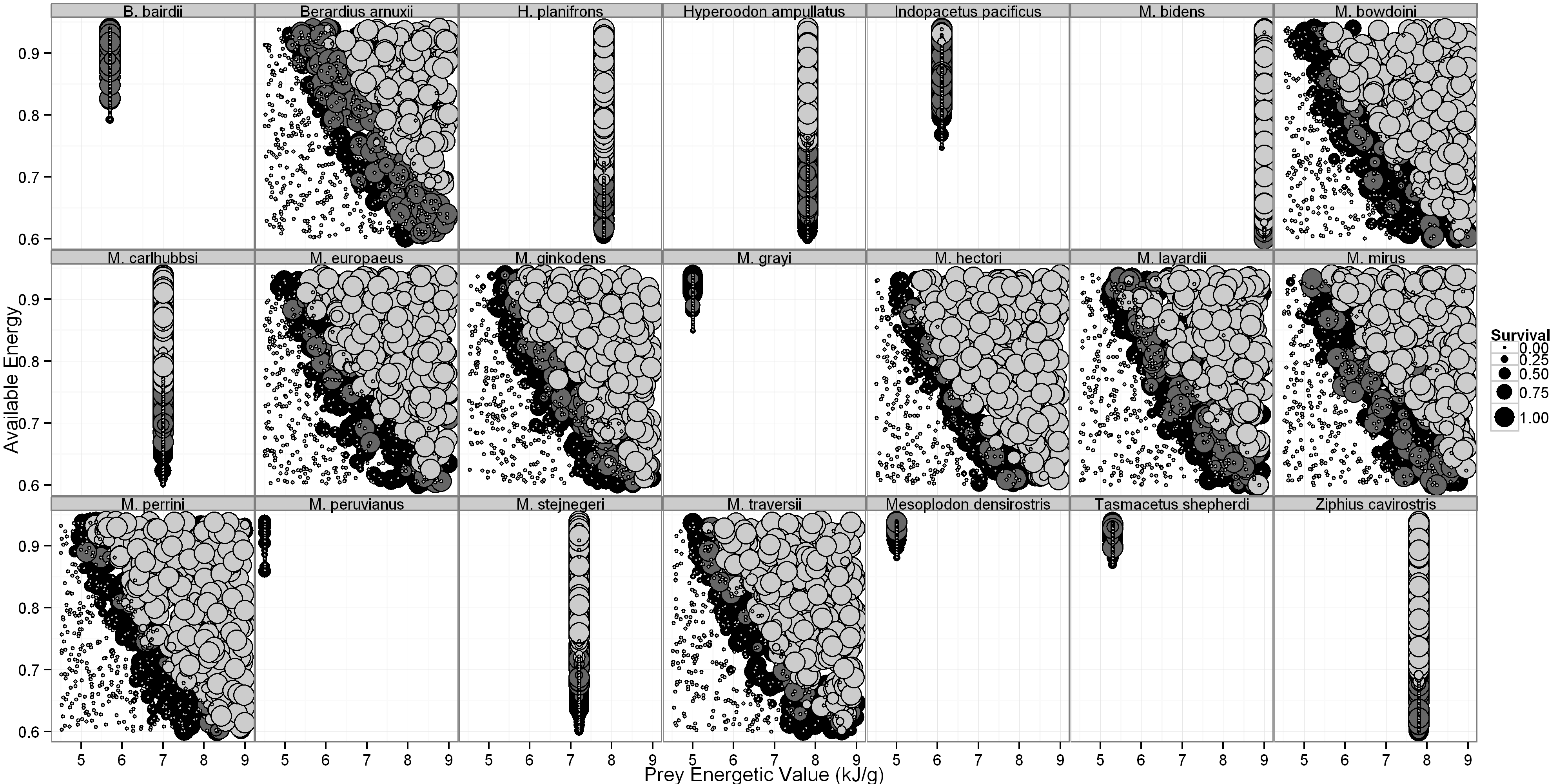

Supplement: Figure S2 — The relationship between the energetic content of prey, available energy and the percentage of adult females (black), calves (dark grey) and fetuses (light grey) in the population to survive, as indicated by the size of the circle. Calves and fetuses can’t survive without their mothers, so adult female survival is not shown when it is equal to that of their offspring. Similarly, if only fetus survival is visible then calf and maternal survival has occurred at the same intensity. Each point is the result from a single simulation. (TIF) [file pone.0068725.s002.tif]

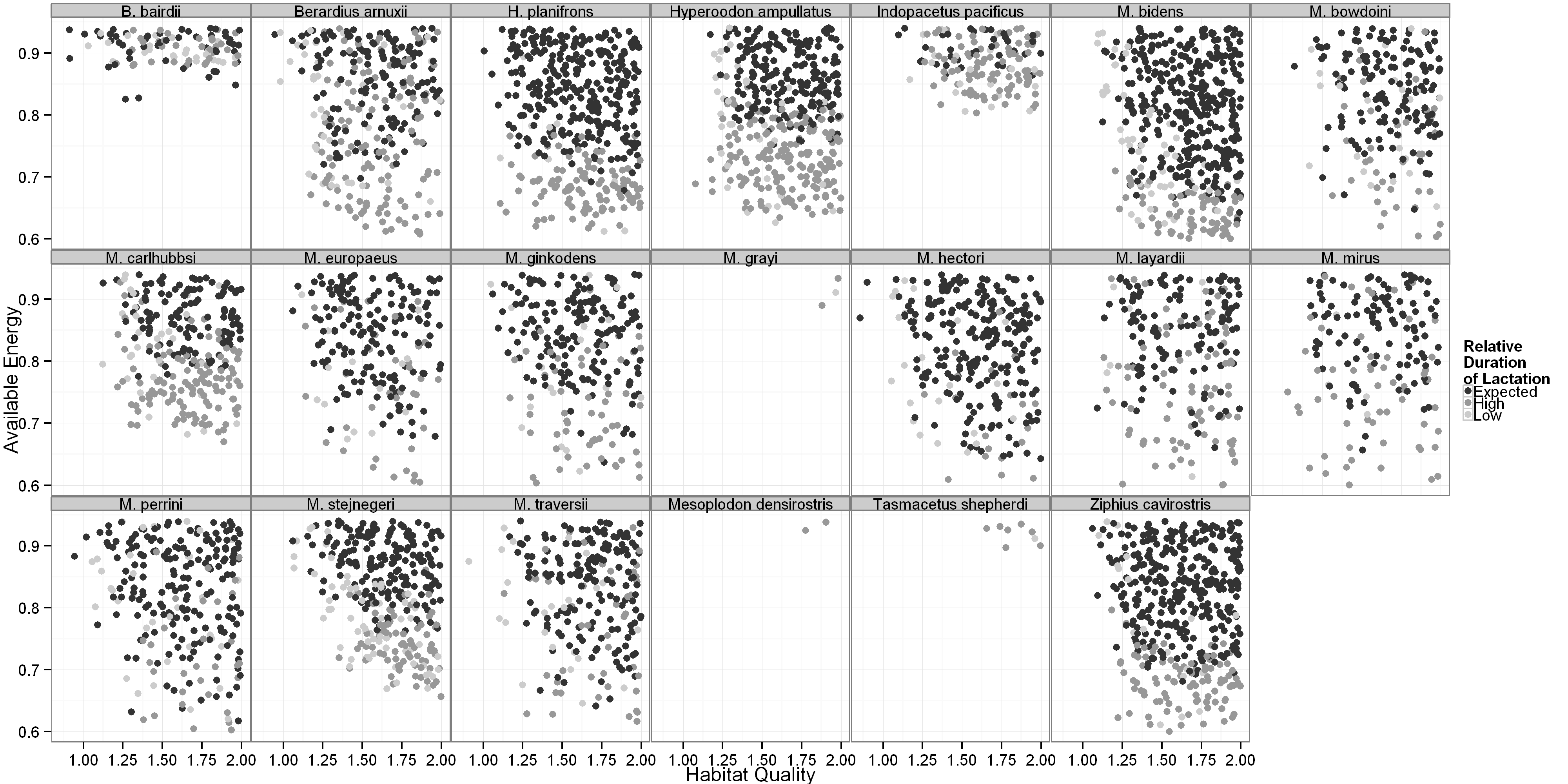

Supplement: Figure S3 — The relationship between available energy, habitat quality and the relative duration of lactation. Light grey dots indicate that the relative duration of lactation was less than expected, which means that not all calves survived to weaning. Black dots indicate that the duration of lactation was equal to the assumed value and dark grey dots indicate that the duration of lactation was longer than expected. M. peruvianus is not shown, since no simulation estimated successfully weaned calves. Each point is the result from a single simulation. (TIF) [file pone.0068725.s003.tif]

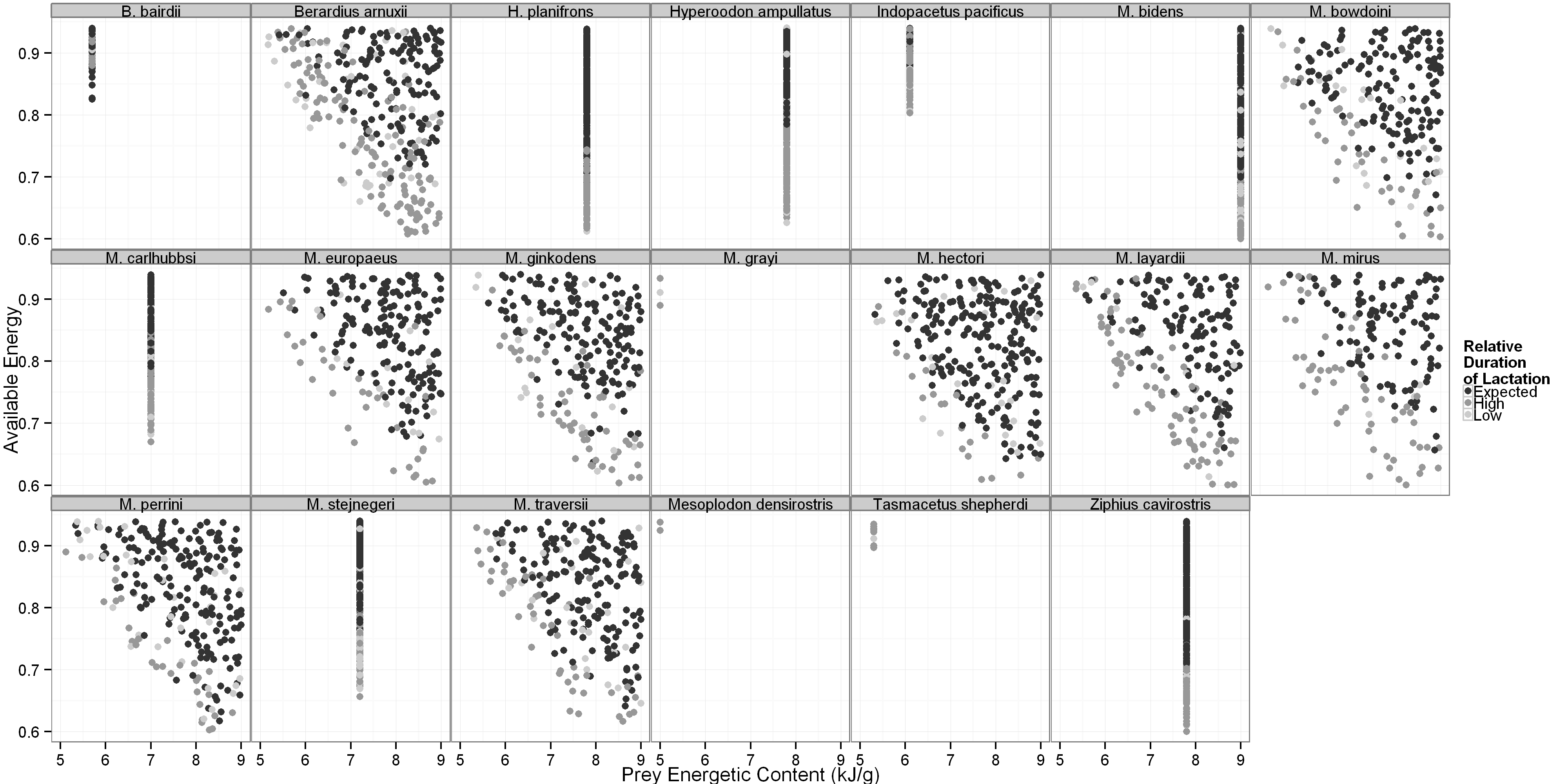

Supplement: Figure S4 — The relationship between available energy, prey energetic content and the relative duration of lactation. Light grey points indicate that the relative duration of lactation was less than expected, which means that not all calves survived to weaning. Black points indicate that the duration of lactation was equal to the assumed value and dark grey points indicate that the duration of lactation was longer than expected. M. peruvianus is not shown, since no simulation estimated successfully weaned calves. Each point is the result from a single simulation. (TIF) [file pone.0068725.s004.tif]
